# Enhanced identification of significant regulators of gene expression

**DOI:** 10.1186/s12859-020-3468-z

**Published:** 2020-04-06

**Authors:** Rezvan Ehsani, Finn Drabløs

**Affiliations:** 1 Department of Mathematics, University of Zabol, Zabol, Iran; 2 Department of Bioinformatics, University of Zabol, Zabol, Iran; 3 Department of Cancer Research and Molecular Medicine, NTNU - Norwegian University of Science and Technology, NO-7491 Trondheim, Norway

**Keywords:** Regulatory impact factor, Gene regulation, Correlated gene expression, Fisher metric, Sobolev metric, Prostate cancer

## Abstract

**Background:**

Diseases like cancer will lead to changes in gene expression, and it is relevant to identify key regulatory genes that can be linked directly to these changes. This can be done by computing a Regulatory Impact Factor (RIF) score for relevant regulators. However, this computation is based on estimating correlated patterns of gene expression, often Pearson correlation, and an assumption about a set of specific regulators, normally transcription factors. This study explores alternative measures of correlation, using the Fisher and Sobolev metrics, and an extended set of regulators, including epigenetic regulators and long non-coding RNAs (lncRNAs). Data on prostate cancer have been used to explore the effect of these modifications.

**Results:**

A tool for computation of RIF scores with alternative correlation measures and extended sets of regulators was developed and tested on gene expression data for prostate cancer. The study showed that the Fisher and Sobolev metrics lead to improved identification of well-documented regulators of gene expression in prostate cancer, and the sets of identified key regulators showed improved overlap with previously defined gene sets of relevance to cancer. The extended set of regulators lead to identification of several interesting candidates for further studies, including lncRNAs. Several key processes were identified as important, including spindle assembly and the epithelial-mesenchymal transition (EMT).

**Conclusions:**

The study has shown that using alternative metrics of correlation can improve the performance of tools based on correlation of gene expression in genomic data. The Fisher and Sobolev metrics should be considered also in other correlation-based applications.

## Background

The transcription of genes is controlled through regulatory processes that are shared between subsets of genes, and this can lead to correlated levels of gene expression within such gene sets [1–3]. For example, increased expression of a gene producing a positive regulator can initiate higher expression values of the downstream genes that are controlled by the regulator, and this leads to correlation in expression values for these genes across a relevant biological process, like a pathway of cellular differentiation. However, this correlation does not by itself show causality. Correlated expression pattern between two genes x and y may be a consequence of interaction between a regulator and a gene being regulated (x → y), but it may also be a consequence of regulatory cascades (x → z → y), or of separate genes being regulated by the same regulator (z → x; z → y). This means that a general correlation network will show all possible interactions between genes, including all the indirect ones, as well as random correlations, making it challenging to identify the most important regulatory interactions in a given system.

The simplest approach for doing a more focused analysis is to specify a potential causality by postulating a specific regulator for a process and hypothesizing that correlated expression levels between the regulator and other genes indicates that this regulator has a significant regulatory impact on downstream targets in the process. This approach has in particular been implemented as regulatory impact factor (RIF) scores [4, 5]. Here transcription factors (TFs) are defined as regulators, and potential downstream targets are identified based on correlated significant differential expression (DE) using two different score values, RIF1 and RIF2. RIF1 identifies factors that are consistently co-expressed with highly abundant DE genes, whereas RIF2 identifies TFs with the ability to act as predictors of the abundance of DE genes.

The RIF approach has been used successfully in several studies. It has been used extensively for analysing data on livestock animals, like cattle and pigs. In such studies the RIF scores have been used to identify key regulators for feed efficiency [6], puberty [7, 8], and intramuscular fat content [9] in cattle, or growth and metabolism [10], high-altitude adaptation [11], and muscle characteristics [12] in pigs, to give a few examples. However, the RIF approach has also been used to analyse human data, in cases as diverse as colorectal cancer [13], effects of melphalan treatment [14], and biomarker candidates for total sleep deprivation [15]. The RIF approach was initially developed for TFs, but there have been a few examples of extension to other classes of regulators, like micro-RNAs (miRNAs) [16] and long non-coding RNAs (lncRNAs) [17], but mainly on data for livestock animals, and with limited testing. There have also been a few examples of integration of RIF scores into other relevant software tools, like RMaNI [18], INsPeCT [19], DCGL [20], RegulatorTrail [21], and REGGAE [22]. There have been some testing and comparisons of RIF to other approaches, as for example in [23, 24]. However, such comparisons can be challenging, at least partly because methods may have different requirements with respect to input data, e.g., gene lists vs. networks [24].

In this project we wanted to explore if the traditional RIF approach could be successfully extended and tested with focus on two important aspects. We have recently shown that correlations in gene expression can be identified more robustly [25] by using alternative correlation metrics like Fisher [26] or Sobolev [27], rather than the standard Pearson or Spearman correlation used in most studies, and we wanted to test if this could be applied to RIF scores. Also, since the initial implementation of RIF scores the interest in other regulators of gene expression has increased, and we wanted to test if specification of genes involved in other regulatory processes could give more insight into the role of these specific regulators. The lncRNAs [28] and genes involved in epigenetic processes (epigenetic factors, EFs) [29] are relevant examples. We therefore made a general implementation for computation of RIF scores that could be combined with alternative correlation metrics and regulators, and tested this implementation on data for prostate cancer, generated by the TCGA Research Network [30]. The study shows that using either the Fisher or the Sobolev metric gives improved identification of relevant genes and gene sets. Specific lncRNAs and epigenetic factors are identified as important for cancer development, in addition to transcription factors.

## Methods

### General workflow

**Fig. 1:**
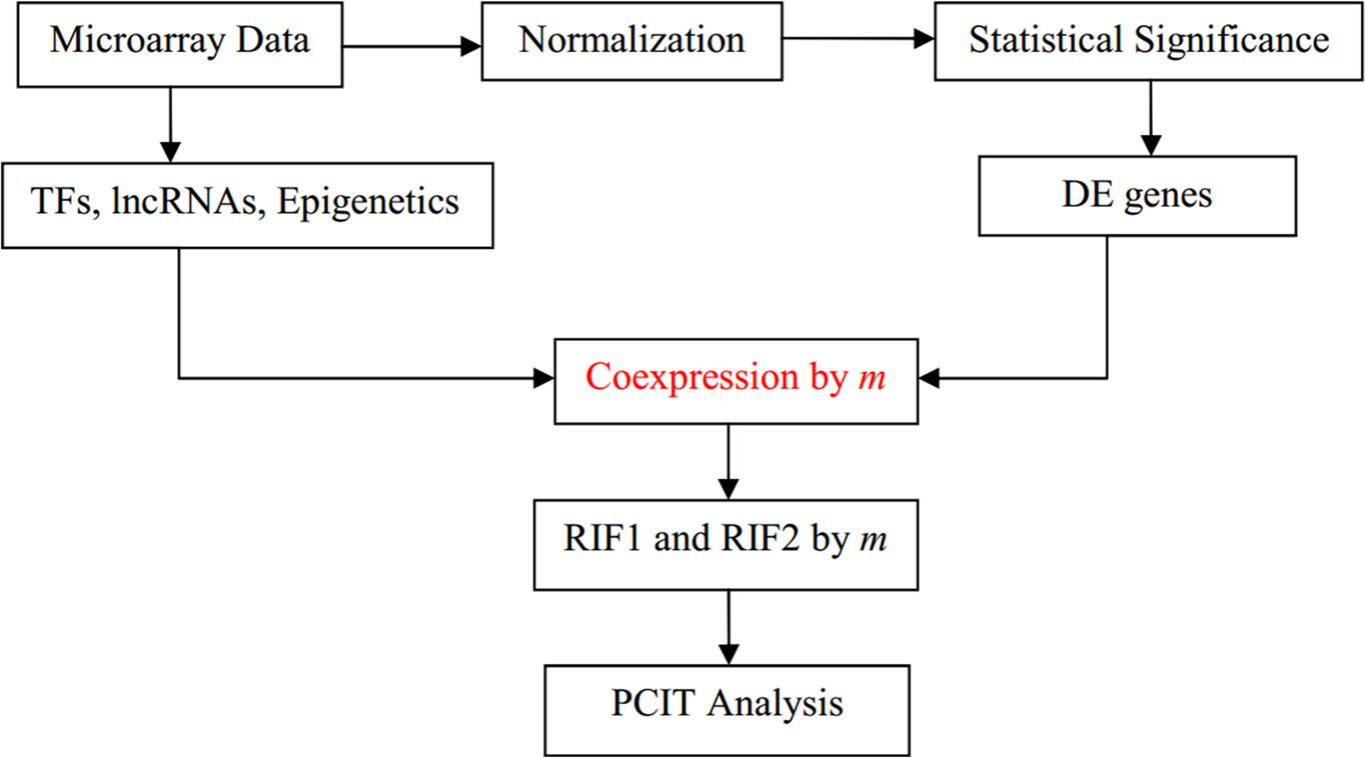
General workflow of the analysis

The schematic workflow is shown in Fig. 1, and uses the general strategy of Reverter et al. [4]. The starting point is a gene expression dataset involving two separate conditions, e.g., microarray data for normal and cancer tissue. The data are normalized, and a standard statistical analysis is used to find target genes with significant difference in expression level between the two conditions, known as differentially expressed (DE) genes. The set of regulators (e.g., TFs, EFs, and lncRNAs) included in the experimental data are extracted, using gene lists from published databases for identification. The correlation of co-expression between regulators and DE genes is computed for the two conditions, and the difference in correlation is used to estimate the differential wiring (DW), as shown below. This can be used to assign RIF scores to the regulators that show consistent differential co-expression with genes that are both highly abundant and differentially expressed (RIF1 score), and to the regulators with the best ability to predict the abundance of DE genes (RIF2 score). In the current project PCIT analysis is subsequently used to find interactions between regulators.

### Datasets

For definition of causality (see Background) we identified three partly overlapping classes of genes representing potential regulators; transcription factors (TFs), epigenetic factors (EFs), and long non-coding RNAs (lncRNAs). We used a list of 1978 TFs from our previous work [31]. We used 815 EFs from the Epifactor database of gene products involved in epigenetics, including 98 TFs [29]. This list also includes some additional well-known regulators, mainly various kinases, here listed together with epigenetic factors. We used 12,980 lncRNAs from Jiang et al. [32].

As a test case we identified key regulators of prostate cancer. We used data generated by the TCGA Research Network [30], using 21 paired samples on prostate adenocarcinoma (PRAD), with count data on gene expression. In each case two samples had been taken from the same donor; one sample to represent Primary Tumor, the other sample representing Solid Tissue Normal.

### Normalization and differential expression

We used the edgeR package [33] to identify differentially expressed (DE) genes. The edgeR is based on a negative binomial model where a weighted fixed mean of the log expression ratios is used to normalize the sequencing depth and gene length between samples. The negative binomial model is made by using expression data where the relation between mean μ and variance ʋ is given by ʋ = μ + αμ^2^. The dispersion factor α is estimated using a combination of common dispersion on all the genes (estimated by a likelihood function), and a gene-specific dispersion (estimated by Bayes method). Finally, an exact test with false discovery rate (FDR) is used to identify DE genes.

### Measures of RIF

For each regulator, the regulatory impact factor (RIF) tries to estimate the change in co-expression between the regulator and the DE genes. The RIF scores have been introduced and described by Reverter et al. [4], where full mathematical definitions can be found. RIF1 identifies regulators that are consistently co-expressed with highly abundant DE genes. It is computed from multiplying phenotype impact factor (PIF) with differential wiring (DW), where PIF is the difference in squared expression for a given DE gene for two conditions, whereas DW is computed from the difference in co-expression correlation between a regulator and the DE gene for the two conditions. Thus, RIF1 captures those regulators that have a large differential wiring to highly abundant highly DE genes. RIF2 identifies regulators with the ability to act as predictors of the abundance of DE genes. It is estimated from the difference of squared expression weighted by the squared co-expression correlation between the regulator and the DE genes in two conditions. The computation of RIF scores was implemented as an R program (see Availability of data and materials).

### Metrics for correlation of co-expression

To identify correlation in co-expression for a typical gene g standard statistical metrics like Pearson and Spearman are normally used. However, in a recent study we used the geometrical metrics Sobolev and Fisher information to annotate lncRNAs based on co-expression [25], and could show that these geometrical metrics had better performance than the more commonly used statistical metrics. A detailed description of each of these novel metrics can be found in [25], which builds on definitions and notations as given by Villman [27] for the Sobolev metric, and definitions and notations given by Lebanon [26] for the Fisher metric.

### Interactions between regulators

We used the PCIT algorithm [34] to find significant interactions between regulators. The gene list used for PCIT included the top 10 regulators with best RIF1_m_ and RIF2_m_ scores for each metric m separately, but only those with a direct and partial correlation ≥0.90 were used for the analysis.

### Analysis of gene sets

For identification of genes previously associated with cancer we used reference data from PubMed, accessed on January 2019. Overlap with existing predefined gene sets was done with the online tool for computing overlap with MSigDB [35, 36]. The tools DAVID [37, 38] and Enrichr [39, 40] were used for enrichment analysis. We used g:Profiler [41] to convert HGNC IDs to UniProt/SwissProt IDs before DAVID analysis. The listed *p*-values for DAVID and Enrichr are after Benjamini correction as done by each of the tools.

## Results

### Important regulators in prostate cancer

**Fig. 2:**
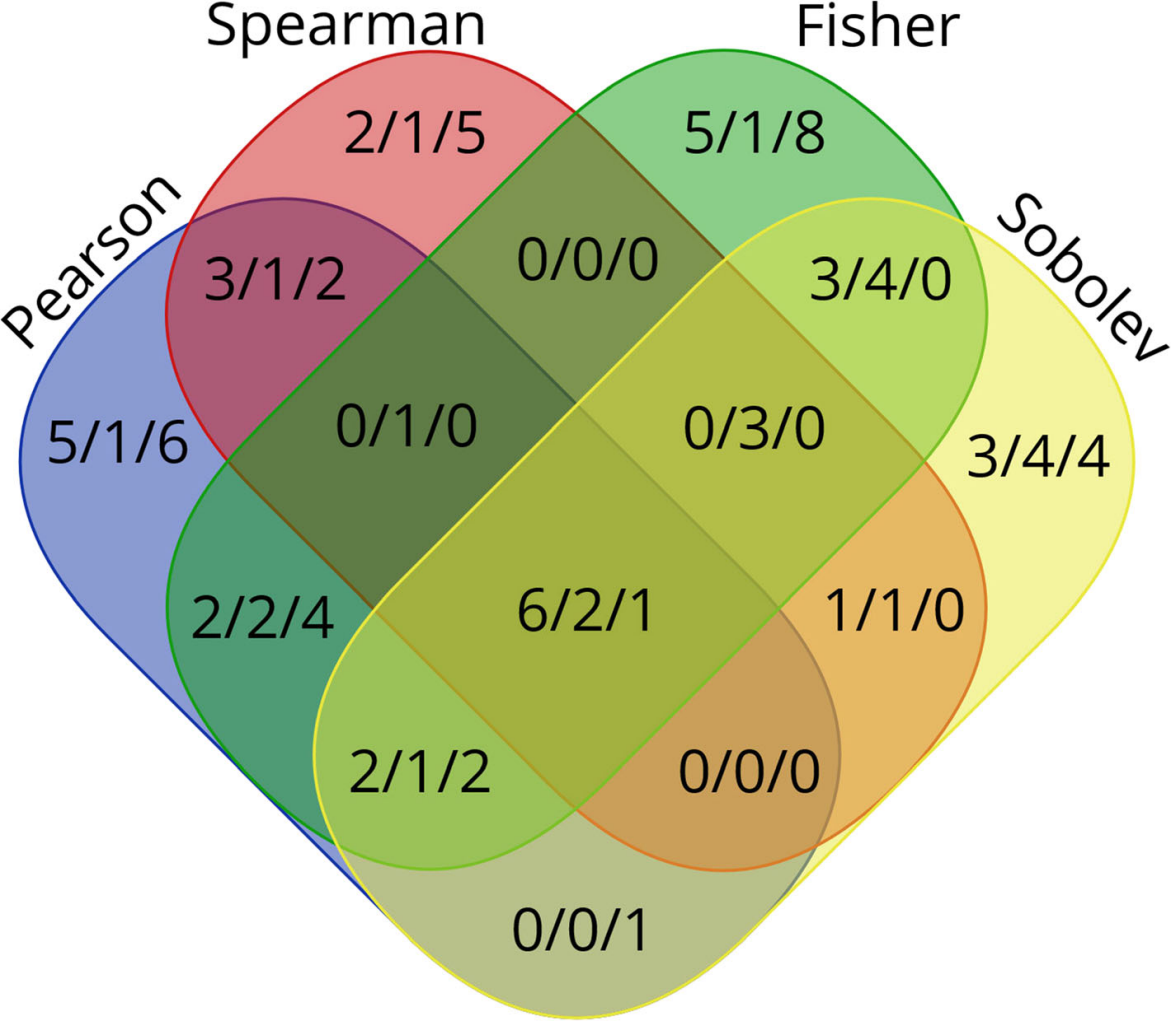
Venn diagram of the significant regulators according to correlation metric. The figure shows numbers for TFs / EFs / lncRNAs, respectively. The Venn diagram tool at [42] was used

Using the data set from TCGA with 21 paired samples on normal and cancerous prostate tissue, the RIF score was computed for all regulators, using external lists of transcription factors, epigenetic factors, and long non-coding RNAs for defining regulators, and four different metrics for correlation of expression values. Significant interactions between regulators were identified by using the PCIT algorithm [34]. The number of significant regulators of each type and metric is shown in Table 1, and the overlap between the results is shown as a Venn diagram in Fig. 2. There is a clear overlap between the different methods, and the largest group of overlaps is for a set of 9 genes found by all correlation metrics. However, in total most entries are unique to each method. This highlights the importance of identifying the most robust correlation measure for subsequent analysis.

**Table 1:** Number of significant regulators for each correlation metric and in total

	Pearson	Spearman	Fisher	Sobolev	Total
TFs	18	12	18	15	32
EFs	8	9	14	15	22
lncRNAs	16	8	15	8	33
Sum	42	29	47	38	87

Total is the number of unique entries across all methods

### Overlap with known key regulators

It seems reasonable to assume that a high-quality prediction should include several important regulators. It may be difficult to define exactly what we mean by “important” in this context, but one approach is to assume that these regulators already have been identified as important, and therefore have been studied (and described) in many publications. We therefore used a simple approach where we counted the number of publications on each gene, as listed in PubMed. This was based on standard PubMed searches using either the gene name itself, or the gene name in combination with “cancer” or “prostate cancer”, and we did this for both transcription factors and epigenetic factors, based on the assumption that these two groups of genes represent the most extensively studied regulators in our study, compared to lncRNAs. The gene for HR (HR lysine demethylase and nuclear receptor corepressor) was not included in this analysis, due to the high number of non-relevant hits during the PubMed searches. The result is shown in Table 2. An extended table with gene names and statistics is available (Additional file 1), as well as tables of gene sets used for the analysis (Additional file 2). The PubMed analysis is a simplistic approach, and it assumes a relatively standardized use of gene names in publications, but it still shows a clear trend, where the alternative metrics Sobolev and Fisher consistently have retrieved the gene sets with most publications, with the Fisher metric doing slightly better than Sobolev.

**Table 2:** Average number of publications in PubMed

	Pearson	Spearman	Fisher	Sobolev
TFs	10.53	4.00	24.82	22.60
EFs	3.75	5.33	21.29	15.20

Numbers are for searching with gene name and “prostate cancer” over all significant regulators

It is possible that this result could be dominated by a small number of highly described genes, where any method retrieving these specific genes (possibly by chance) would get a high score. We therefore identified the genes that were most frequently described in combination with prostate cancer, for TFs (8 genes) and EFs (7 genes), i.e., 15 genes in total. We then checked how many of these genes were found by each method, which was 5 genes for Pearson, 3 genes for Spearman, 11 genes for Fisher, and 8 genes for Sobolev. This again shows that the Fisher and Sobolev metrics gave the best performance with respect to identification of well-known regulators.

### Overlap with predefined gene sets

**Fig. 3:**
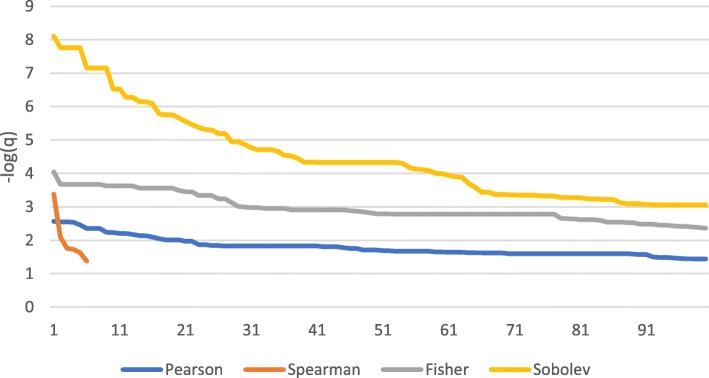
Significance of overlap with MSigDB gene sets. The graph shows ranked FDR q-scores (as -log) of overlap between each gene set and the MSigDB reference sets

It also seems reasonable to assume that a good set of regulators should be informative by showing significant overlap with predefined gene sets from literature. Several such gene sets have been collected in the MSigDB database, which also offers a web-based tool for computing overlap between input gene sets and gene sets in the MSigDB database. This was used to estimate the overlap between the gene set for each of the methods against all gene sets in MSigDB, except gene sets based on GO terms. GO terms were excluded because the initial set of genes is based on their role in gene regulation (like transcription factors), therefore all GO terms related to gene regulation will be highly significant, which will then dominate the analysis, whereas in this case we want to focus on what we can learn *in addition* to what we already know. For each gene set (Pearson, Spearman, Fisher, Sobolev) we retrieved the FDR q-score for the overlap between the sets and transformed it to its -log value for all significant overlaps (limited to the top 100). The results are shown in Fig. 3. Again, the results show that Fisher and Sobolev is better than Pearson and Spearman, as those metrics define gene sets that show a more significant overlap with existing gene sets from literature.

**Fig. 4:**
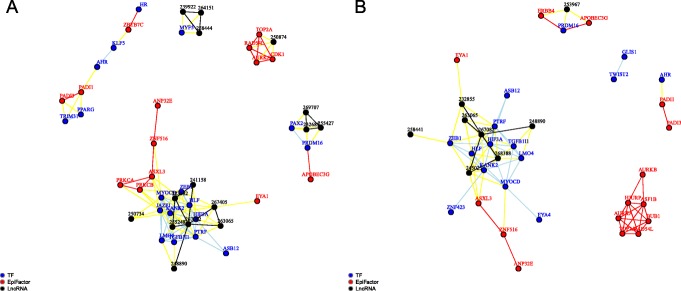
Network of interactions between significant regulators in prostate cancer. The correlations have been computed according to the Fisher metric (**a**) and the Sobolev metric (**b**), and the network nodes of significant regulators are TFs (blue), EFs (red), and lncRNAs (black, with ENSG numbers as ENSG00000xxxxxx)

We will now discuss the results in more detail. Based on the results regarding metrics we will focus on regulators identified by the Fisher and Sobolev metrics, and in particular clusters of regulators that could be found in both analyses. Figure 4 show networks of significant regulators and interactions in prostate cancer as estimated with these two metrics.

## Discussion

### Overlap with specific MSigDB gene sets

The overlap of sets of regulators with specific MSigDB gene sets, including overlap with the GO sets, can provide interesting information on relevant processes that were identified by the analysis. As expected, both sets (Fisher and Sobolev) showed significant overlap with gene sets related to gene regulation, like GO_POSITIVE_REGULATION_OF_GENE_EXPRESSION. However, the most significant terms for Sobolev were GO_CHROMOSOME_ORGANIZATION and GO_CHROMATIN_ORGANIZATION, indicating a strong involvement of chromatin-specific processes. The original analysis (without including the GO-specific gene sets) had best significance for Sobolev against GNF2_BUB1B (Neighborhood of BUB1B, which is an important kinase involved in the mitotic spindle checkpoint [43], ROSTY_CERVICAL_CANCER_PROLIFERATION_CLUSTER, and MODULE_54 (Cell cycle expression cluster). The overlap with the cell cycle expression cluster consisted of the genes TOP2A, BUB1, AURKA, AURKB, HJURP, ASF1B, ANP32E and RAD54L. Most of these genes were also important for the other overlaps with high significance, and several of the genes will be discussed below. The overlap for the Fisher gene set also included several interesting gene sets, like BURTON_ADIPOGENESIS_3 (Differentiation into adipocytes), and involved in particular the genes CDK1, TOP2A, and AURKA.

### Enrichment analysis

Properties represented by the sets of regulators can also be described by enrichment analysis. We used DAVID and Enrichr as two complementary approaches. Both do standard enrichment analysis, but DAVID can also do clustering on enrichment results, which can highlight interesting trends across the individual enrichments, whereas Enrichr has a very large set of reference libraries, which can highlight additional enriched properties.

In DAVID the gene sets from both Fisher and Sobolev gave clusters with strong enrichment for processes associated with transcription regulation, as expected, and clusters with strong enrichment for zinc fingers. In addition, the Sobolev gene set gave clusters with enrichment for terms associated with cell cycle, centromere and chromosome, including cell proliferation, mitosis and cell division. This trend was reinforced by enrichment results for GOTERM_CC_FAT, where significant enrichments included terms associated with centromeric region (*p* = 1.3e-2), centrosome (*p* = 8.8e-2), and spindle (8.8e-2). The gene set retrieved by using the Sobolev metric therefore highlights the importance of fundamental processes directly associated with cell division.

The Enrichr analysis showed a strong enrichment for downregulated genes from GEO data on prostate cancer (*p* = 3.6e-5), as expected. Enrichr also highlighted knockdown data from LINCS L1000 Kinase Perturbations for WEE1 (p = 8.8e-4) and ERBB3 (*p* = 4.3e-4). Especially WEE1 (WEE1 G2 checkpoint kinase) is known to be an important cell cycle checkpoint kinase [44], but also ERBB3 (Erb-B2 receptor tyrosine kinase 3) is important in cancer development and has been identified as a potential target for treatment (e.g. [45],). There was also a significant enrichment for FOXM1 ChEA 2016 ChIP-Seq data (*p* = 2.9e-2) and E2F4 ENCODE data (*p* = 7.3e-4), and both FOXM1 (Forkhead box M1) and E2F4 (E2F transcription factor 4) are known to be important for cell cycle regulation, see for example [46]. This highlights again the central role of cell cycle regulation in cancer development.

### Specific gene clusters and individual genes

The computational analysis also identified potential interactions between genes, leading to a network-like representation of clusters of interacting genes, indicating genes with shared activities. These clusters can be seen in Fig. 4. Specifically, there is a highly interacting set of genes consisting of TOP2A, RAD54L and AURKA, possibly with the inclusion of CDK1, AURKB, HJURP, ASF1B, and BUB1 (here discussed as gene set A). There is also a more diverse set, but with a very high overlap between Fisher and Sobolev consisting of EYA1, ZEB1, ASB12, PTRF, HIF3A, HLF, TGFB1I1, LMO4, MYOCD, ASXL3, ZNF516, and ANP32E, possibly with the inclusion of ZNF423, EYA4, PRKCA, PREKCB, JAZF1 and KANK2 (gene set B). Many of these genes represent well-known cases of regulators involved in cancer development, including prostate cancer, as can be seen from Additional file 1, although at different levels of experimental verification. The more novel predictions obviously must be experimentally verified, but the relevance of individual cases (based on the PubMed data) indicates that many of the predictions are likely to be true positive. This is particularly relevant for the lncRNAs, where in most cases very little is known about their function.

Gene set A overlaps with cell cycle signatures [47, 48], including the MSigDB cell cycle signature mentioned above, confirming that this gene set represents cell cycle regulation. The individual genes are known to be involved in several processes, including processes related to DNA topology, centrosomes and mitotic spindle formation. This includes AURKA (Aurora kinase A), which is associated with centrosome maturation and separation, and regulates spindle assembly and stability. It has been shown that AURKA is important for development of prostate cancer, and that it represents a possible target for treatment [49]. TOP2A (DNA Topoisomerase IIα) controls and alters the topological states of DNA and is important for proper segregation of daughter chromosomes [50]. RAD54L (RAD54 Like) is a helicase which seems to influence DNA topology in different ways, e.g., in chromatin remodelling, homologous recombination and interaction with Holliday junctions [51]. The Fisher gene set also includes CDK1 (Cyclin dependent kinas), which is known to be essential for cell division, and is known to be involved in regulation of cell cycle through centrosomes / spindle assembly [52]. The Sobolev gene set also includes ASF1B (Anti-silencing function 1B histone chaperone), which is essential for the mitotic spindle checkpoint during the cell cycle [53], AURKB (Aurora kinase B), which participates in the regulation of alignment and segregation of chromosomes [54], HJURP (Holliday junction recognition protein), which mediates the centromere-specific assembly of CENP-A nucleosomes important for chromosome segregation [55, 56], and BUB1 (Mitotic checkpoint serine/threonine kinase), which is essential for spindle-assembly checkpoint signalling [57]. This is a strong indication that cell cycle processes associated with chromosome handling and the mitotic spindle are important in prostate cancer.

Gene set B is more diverse and does not show very strong overlap with any specific gene set in MSigDB. However, many of the genes in gene set B are known to be involved in the endothelial to mesenchymal transition (EMT), which is a key process in prostate cancer. ZEB1 (zinc finger E-box binding homeobox 1) has frequently been associated with cancer in general, and prostate cancer in particular. It has been shown that up-regulation of ZEB1 drives EMT in human prostate cancer cells [58]. HIF3A (hypoxia inducible factor 3 subunit alpha) is another gene that has been linked to prostate cancer. It is a negative regulator of HIF1A [59], and it has been shown that destabilisation of HIF1A by ERβ can induce EMT [60]. EYA1 (EYA Transcriptional coactivator and phosphatase 1) is a transcriptional coactivator of the SIX homeobox genes and is a coregulator of TGF-β signalling during EMT [61]. LMO4 (LIM domain only 4) has been shown to be an essential cofactor in EMT at least in neuroblastoma and neural crest cells [62]. ANP32E (Acidic nuclear phosphoprotein 32 family member E) is a histone chaperone that mediates removal of histone H2A.Z from the nucleosome [63], and it has been shown that H2A.Z is a master regulator of EMT [64]. TGFB1I1 (Transforming growth factor beta 1 induced transcript 1) is a coactivator of the androgen receptor, it is known to be associated with prostate cancer, and it can induce EMT, at least in astrocytomes. From the Fisher gene set, JAZF1 (JAZF zinc finger 1) has been shown to promote prostate cancer progression through JNK/Slug, leading to enhanced EMT [65]. Although these genes are just examples of genes identified by our analysis and the processes they may be involved in, this list seems to be a clear confirmation of the importance of EMT in prostate cancer [66].

Interestingly, gene set B also includes 3 lncRNAs that were found by both Fisher and Sobolev, consisting of ENSG00000263065, which is antisense to exon 21 and 22 of MYH11, ENSG00000248890, which is antisense to HHIP, close to TSS, and ENSG00000267082, which is antisense to DOCK6. MYH11 (Myosin heavy chain 11) has been used as a marker of mesenchymal and endothelial differentiation [67], and HHIP (Hedgehog interacting protein) can be linked to EMT through the hedgehog pathway [68]. Although these associations between lncRNAs and cancer are more circumstantial, they are consistent with the general view that EMT is important in prostate cancer.

We have compared the gene sets from this study to other sets of “cancer genes”, using the library of 299 cancer driver genes of Bailey et al. [69], the COSMIC library of 723 cancer genes [70], and the knockout library of 684 genes of Hart et al. [71]. In all cases the overlap was very low, in the range of just 1–2 genes. However, this is hardly surprising, as they represent quite different classes of cancer genes. The cancer genes are genes where a mutation may drive cancer, possibly without changing the expression level of the gene itself. The knockout experiments, on the other hand will identify genes that are essential to survival of the cancer cell, but not necessarily linked to changes in expression level. The RIF approach focuses on regulators where it is possible to observe a change in expression level, linked to cancer, and where this change is reflected in a downstream process, possibly regulated by the regulator. This means that we identify parts of the regulatory system that are affected by the changes introduced through cancer, rather than the cancer genes themselves, and the overlap between these different approaches will therefore be low.

## Conclusions

We have developed an extended approach for identification of important regulators in biological processes, based on correlations in gene expression. We have implemented alternative metrics for correlation and used this on additional groups of regulators; epigenetic factors and lncRNAs. There is no benchmark dataset for this type of analysis, which makes it difficult to compare our extensions to previous approaches. However, we have shown that using alternative metrics as correlation measures identifies more genes that previously have been associated with cancer and identifies gene sets with better overlap with known gene sets, using prostate cancer as a test case. A case-by-case study of the genes and gene sets shows that the identified genes are relevant for understanding the processes involved in development of prostate cancer, and that they point at mitotic spindle formation and the endothelial to mesenchymal transition as important processes. The study also identifies specific lncRNAs that are likely to be important in these processes.

## Supplementary information

**Additional file 1.** Gene lists with PubMed statistics.

**Additional file 2.** Gene sets used for enrichment analysis.

**Additional file 3.** Identifiers for TCGA datasets on prostate cancer as downloaded from the GDC data portal.

## Data Availability

The Cancer Genome Atlas (TCGA) datasets for prostate cancer supporting the conclusions of this article are available in the repository in Genomic Data Commons Data Portal at https://portal.gdc.cancer.gov/. The list of datasets is available in Additional file 3. The software for computing RIF scores with alternative correlation measures is available via GitHub at [72]. Project name: RIF scores with alternative correlation measures. Project home page: https://github.com/RezvanEhsani Archived version: Please see the GitHub repository. Operating system(s): Platform independent. Programming language: R. Other requirements: Please see the GitHub repository. License: GNU GPL. Any restrictions to use by non-academics: None.

## Abbreviations

DE: Differential expression
DW: Differential wiring
EF: Epigenetic factor
EMT: Epithelial-mesenchymal transition
FDR: False discovery rate
GO: Gene ontology
lncRNA: Long non-coding RNA
PIF: Phenotype impact factor
RIF: Regulatory impact factor
TF: Transcription factor
